# Association between early ICU fever burden and 28-day mortality in patients with acute ischemic stroke: a retrospective cohort study based on the MIMIC-IV database

**DOI:** 10.3389/fneur.2026.1900723

**Published:** 2026-08-20

**Authors:** Caifeng Ma, Xin Zheng, Shuo Tang, Yifan Mo, Xu Wang, Jia Zeng, Ya Gao, Xiangtian Liu, Xinghan Tian

**Affiliations:** 1 Department of Intensive Care Medicine, The Affiliated Yantai Yuhuangding Hospital of Qingdao University, Yantai, China; 2 The Second Clinical Medical School, Shandong Medical and Pharmaceutical University, Yantai, China; 3 Shandong Second Medical University, Weifang, China; 4 Yantai Yuhuangding Hospital, Yantai, Shandong, China

**Keywords:** acute ischemic stroke, fever, fever burden, ICU, MIMIC-IV

## Abstract

**Background:**

Fever is common during the acute phase of acute ischaemic stroke and has been associated with poor outcomes. However, single temperature values may not adequately capture the cumulative effect of sustained thermal exposure. This study evaluated the association between early ICU fever burden and 28-day all-cause mortality in patients with acute ischaemic stroke.

**Methods:**

This retrospective cohort study used data from the MIMIC-IV database. Adult patients with acute ischaemic stroke admitted to the ICU for the first time were included. Fever burden was calculated during the first 72 h after ICU admission as the area under the temperature–time curve above 37.9 °C and was standardized to 24 h. Patients were classified into no, low and high fever-burden groups. The primary outcome was 28-day all-cause mortality from ICU admission. Cox proportional hazards models with multiple imputation were used for the primary analysis. Sensitivity analyses, inverse probability of treatment weighting and subgroup analyses were performed to assess the robustness of the findings.

**Results:**

A total of 3,016 patients were included, of whom 2,229 had no fever burden, 394 had low fever burden and 393 had high fever burden. The 28-day mortality rates were 15.3, 28.7 and 36.6%, respectively. In the fully adjusted multiply imputed Cox model, low and high fever burden were associated with higher 28-day all-cause mortality compared with no fever burden, with adjusted HRs of 1.404 (95% CI 1.126–1.751) and 1.948 (95% CI 1.564–2.427), respectively. The association was generally supported by complete-case analysis, IPTW analysis, alternative exposure windows, landmark analyses, alternative temperature thresholds, adjustment for temperature measurement density and exclusion of patients with sepsis.

**Conclusion:**

In ICU patients with acute ischaemic stroke, greater fever burden during the first 72 h after ICU admission was associated with higher 28-day all-cause mortality. Early ICU fever burden may provide time-integrated prognostic information, but its clinical utility and therapeutic implications require further prospective validation.

## Introduction

Acute ischemic stroke (AIS) is one of the leading causes of death and long-term disability worldwide (1). Although most patients with mild-to-moderate AIS can receive standardized treatment in stroke units, approximately 15 to 20% of patients still rapidly deteriorate because of secondary complications, such as a decreased level of consciousness, severe neurological deficits, or cerebral edema (2–4). Cerebral oedema is an important cause of neurological deterioration after ischaemic stroke and may contribute to poor outcomes (5). In the setting of severe ischemic brain injury, fever is a common secondary physiological abnormality during the acute phase of AIS. Its occurrence is rarely attributable to a single factor; rather, it may be related to stroke-induced autonomic dysregulation, impaired thermoregulation, systemic inflammatory and immune changes, and infectious complications such as stroke-associated pneumonia (4, 6, 7). Previous studies have shown that fever is common in patients with AIS, with approximately one-third developing varying degrees of temperature elevation in the early stage of the disease. This phenomenon has been closely associated with a higher risk of death and worse neurological outcomes (5). Ischemic brain tissue is particularly vulnerable to elevated temperature. Prolonged heat exposure increases cerebral metabolic demand, worsens excitotoxic injury, amplifies neuroinflammation, and disrupts the blood–brain barrier, thereby accelerating energy failure in the ischemic microenvironment and exacerbating irreversible brain injury (8, 9).

Although post-stroke fever is widely recognized as being associated with adverse outcomes, risk stratification in previous studies has largely depended on either the single maximum temperature (*Tmax*) or a binary definition of fever (10). Such static metrics capture temperature at specific time points only and do not fully reflect its persistence or cumulative burden (11). To better characterize cumulative exposure to elevated temperature, recent studies have increasingly introduced the concept of “fever burden,” which refers to the time integral of temperature elevations above a predefined threshold, typically expressed in °C-hours (12, 13). Previous studies suggest that this metric may more effectively capture the overall burden of sustained thermal exposure than a single peak temperature (13). However, studies focusing on ICU patients with AIS remain limited. Therefore, the association between cumulative fever exposure and adverse outcomes in critically ill patients with AIS warrants further investigation.

Therefore, this study aimed to evaluate the association between early ICU fever burden during the first 72 h after ICU admission and 28-day all-cause mortality in critically ill patients with AIS based on the MIMIC-IV database. By quantifying cumulative temperature exposure, this study may improve early risk stratification and provide a basis for future prospective studies on temperature management.

## Methods

### Data source

This was a single-center retrospective cohort study based on the Medical Information Mart for Intensive Care IV (MIMIC-IV v3.1), a publicly available critical care database in the United States (14). The MIMIC-IV database was developed jointly by the Laboratory for Computational Physiology at the Massachusetts Institute of Technology (MIT) and Beth Israel Deaconess Medical Center (BIDMC). It contains electronic health records (EHRs) of critically ill patients, including vital signs, laboratory measurements, medication records, and outcome data. The development and use of the MIMIC-IV database were approved by the Institutional Review Boards (IRBs) of both MIT and BIDMC. Because the database has been fully de-identified in accordance with the Health Insurance Portability and Accountability Act (HIPAA) Safe Harbor standards, informed consent was waived. Data extraction was performed in accordance with the data use agreement, and the researchers completed the required ethical training and obtained database access authorization. Specifically, author Xiangtian Liu was granted access to the database (Certification ID: 65112747).

### Study population

**Figure 1:**
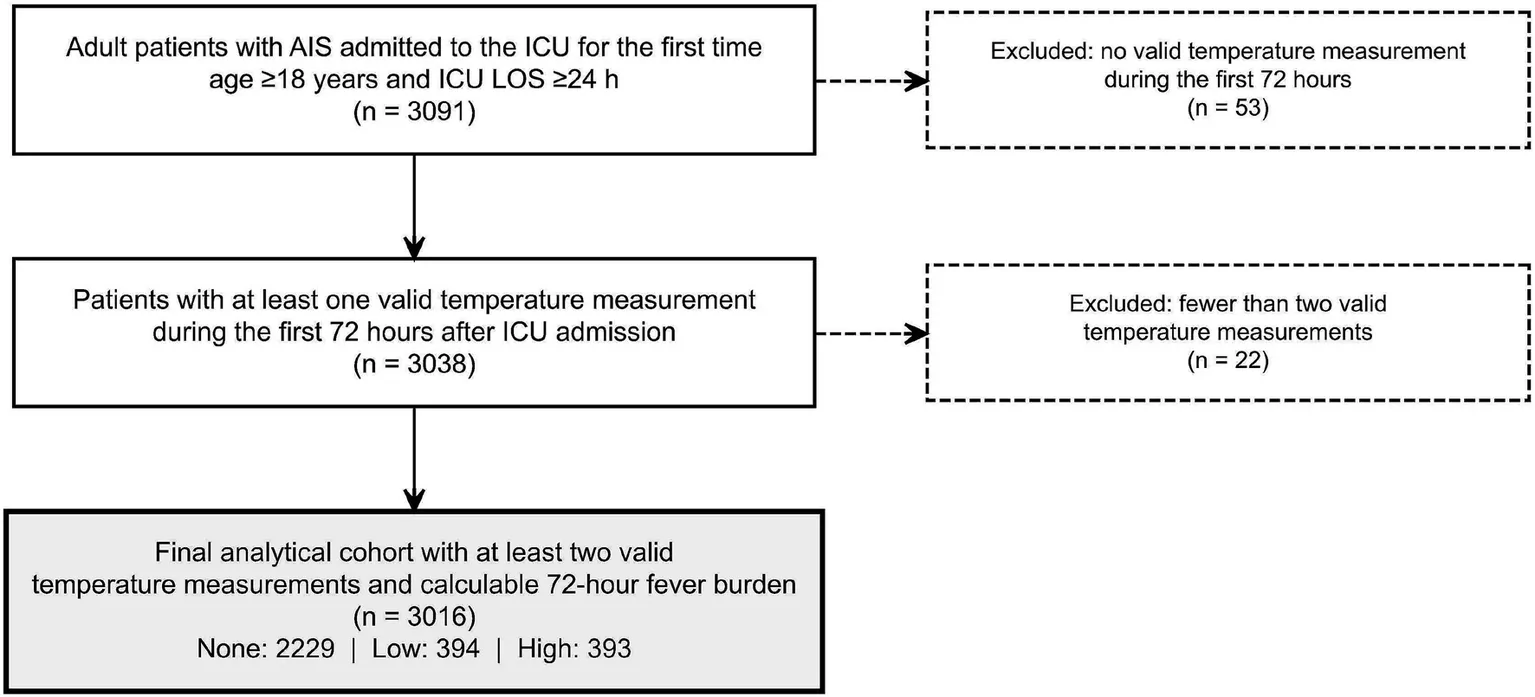
Flowchart of patient selection.

Patients with acute ischaemic stroke (AIS) were identified from MIMIC-IV using International Classification of Diseases, Ninth and Tenth Revision (ICD-9 and ICD-10) diagnosis codes. Eligible patients were adults aged ≥18 years who were admitted to the ICU for the first time and had an ICU length of stay of at least 24 h. When a patient had more than one hospitalization or ICU stay, only the first ICU admission was included. Patients were excluded if they had no valid temperature measurements during the first 72 h after ICU admission or fewer than two valid temperature measurements within this period. The cohort selection process is shown in Figure 1.

### Fever burden calculation and grouping

The primary exposure was time-normalized fever burden during the first 72 h after ICU admission. Body temperature values were converted to degrees Celsius. A temperature above 37.9 °C was defined *a priori* as the fever threshold (13). Temperature measurements recorded within the first 72 h after ICU admission were extracted for each patient. Fever burden was calculated as the area under the temperature curve above the predefined threshold (13). For a continuous temperature trajectory, cumulative fever burden was defined as:


$$\text{Fever Burden}=\int_{t_{0}}^{t_{3}}\max(T(t)-T_{\text{threshold}},0)\;\mathrm{dt}$$

Here, *T(t)* represents the continuous temperature at time point t, $T_{th\mathrm{res}h\mathrm{old}}$ is the predefined threshold of 37.9 °C. Since temperature measurements in electronic health records are discrete and irregularly spaced, the trapezoidal rule was used to approximate the area above the threshold between adjacent measurements:


$$\text{Fever Burden}\approx \sum_{i=1}^{n-1}\frac{\begin{array}{l} \max(T_{i}-T_{\text{threshold}},0)\\ +\max(T_{i+1}-T_{\text{threshold}},0) \end{array}}{2}\times (t_{i+1}-t_{i})$$

Here, T*_i_* and *T_i + 1_* are consecutive temperature values, and *t_i + 1_ − t_i_* is the time interval in hours. Cumulative fever burden was expressed in °C-hours.

To account for differences in observation time within the 72-h window, cumulative fever burden was divided by the observed hours and standardized to 24 h. Observed hours were defined as the elapsed time between the first and last valid temperature measurements within the 72-h window. This time-normalized value was defined as the daily mean fever burden and expressed as °C-hours per 24 h. Patients with a daily mean fever burden of 0 were classified as the no fever-burden group. Among patients with positive daily mean fever burden, the median value was used to define the low and high fever-burden groups. The cutoff value used to distinguish the low and high fever-burden groups was 0.6 °C-hours/24 h.

### Variable extraction

Extracted variables included demographic characteristics, comorbidities, early ICU physiological variables, laboratory measurements, illness-severity scores, organ-support variables, infection-related variables and temperature monitoring variables. Demographic characteristics included age at ICU admission, sex and race. Comorbidities included diabetes mellitus, hypertension, coronary heart disease, atrial fibrillation, chronic pulmonary disease, renal disease, liver disease, malignancy and components of the Charlson Comorbidity Index. Early ICU physiological variables included heart rate, blood pressure, respiratory rate, peripheral oxygen saturation and body temperature. Laboratory variables included white blood cell count, hemoglobin, platelet count, serum creatinine, blood urea nitrogen, electrolytes, glucose, coagulation indices and international normalized ratio. Illness severity was assessed using the Sequential Organ Failure Assessment score and the Simplified Acute Physiology Score II. Organ-support variables included mechanical ventilation and haemopurification or renal replacement therapy. Sepsis was extracted as an infection-related variable. Temperature monitoring variables included the number of temperature measurements and temperature measurement density during the first 72 h. Covariates were selected according to clinical relevance, data availability and the directed acyclic graph shown in Supplementary Figure 1. The extent of missing data is shown in Supplementary Table 1.

### Outcome definition

The primary outcome was 28-day all-cause mortality from ICU admission. Survival time was calculated from the time of ICU admission to death or administrative censoring at 28 days. Patients who were alive beyond 28 days were censored at day 28.

### Statistical analysis

Patients were grouped according to time-normalized fever burden during the first 72 h after ICU admission. Continuous variables are presented as median [interquartile range], and categorical variables as *n* (%). Group differences were assessed using the Kruskal-Wallis test, chi-square test, or Fisher exact test, as appropriate.

Kaplan–Meier curves and the log-rank test were used to compare unadjusted 28-day survival across fever-burden groups. Cox proportional hazards models were used to estimate hazard ratios and 95% confidence intervals for 28-day all-cause mortality, with the no fever-burden group as the reference. The extent of missing data is shown in Supplementary Table 1. Covariates with more than 50% missingness were not included in the primary Cox models. Missing covariate data in the primary Cox analysis were handled using multiple imputation by chained equations with random forest imputation. The exposure variable, outcome indicator and follow-up time were not imputed. Twenty imputed datasets with 20 iterations were generated. Convergence of the multiple imputation procedure was assessed using trace plots, which are shown in Supplementary Figure 2. Cox estimates from the imputed datasets were pooled using Rubin’s rules.

Sequential Cox models were fitted. The crude model included fever-burden group only. Model 1 adjusted for demographic characteristics and major comorbidities. Model 2 further adjusted for early ICU severity, vital signs and laboratory variables. Model 3 additionally included organ-support variables. Covariates were selected according to clinical relevance, data availability and the directed acyclic graph, without stepwise selection. The proportional hazards assumption was assessed using Schoenfeld residuals in complete-case Cox models, and the results are presented in Supplementary Table 2. Variables showing evidence of non-proportionality were handled using stratification where appropriate. Multicollinearity was assessed using the variance inflation factor. Sensitivity analyses included complete-case Cox regression, adjustment for temperature measurement density, 24-h and 48-h fever-burden windows, day-3 and day-7 landmark analyses, alternative fever thresholds, and exclusion of patients with sepsis. Inverse probability of treatment weighting was performed as a robustness analysis using complete-case data. A multinomial propensity-score model was used for the three fever-burden groups. Stabilized weights were truncated at the 1st and 99th percentiles, and covariate balance was assessed using standardized mean differences. IPTW-weighted Cox regression and weighted Kaplan–Meier curves were used to examine the consistency of the findings. Prespecified subgroup analyses were performed according to age, sex, race and selected comorbidities.

All analyses were performed using R software, with the survival, survminer, mice, nnet, survey, cobalt, ggplot2 and compareGroups packages. A two-sided *p* value <0.05 was considered statistically significant.

## Results

### Baseline characteristics

Among 3,016 eligible ICU patients with acute ischaemic stroke, 2,229 (73.9%) had no fever burden during the first 72 h after ICU admission, 394 (13.1%) had low fever burden, and 393 (13.0%) had high fever burden. Among patients with positive fever burden, the median daily mean fever burden used to define the low and high fever-burden groups was 0.6 °C·h/24 h. Patients in the high fever-burden group were younger and more frequently male. The median age was 65.8 years in the high-burden group, compared with 70.4 years in the low-burden group and 72.5 years in the no-burden group. The proportions of men were 61.6, 49.5, and 48.2%, respectively.

Several indicators of acute illness differed across the fever-burden groups. Sepsis was present in 80.2% of patients in the high-burden group, compared with 59.9% in the low-burden group and 28.6% in the no-burden group. Mechanical ventilation was also more frequent among patients with fever burden. By contrast, the Charlson Comorbidity Index and the prevalence of major chronic cardiovascular comorbidities showed less marked variation across groups.

During the first 72 h of ICU admission, patients with higher fever burden generally had higher white blood cell counts, heart rates, respiratory rates, glucose concentrations, and recorded temperatures. The number and density of temperature measurements were also higher in the high-burden group, indicating differences in monitoring intensity across groups (Table 1).

**Table 1:** Baseline characteristics by early ICU fever-burden group.

Characteristic	No fever burden (*n* = 2,229)	Low fever burden (*n* = 394)	High fever burden (*n* = 393)	*p* value
Demographic characteristics				
Age, years	72.5 [61.0, 82.4]	70.4 [60.6, 81.7]	65.8 [54.1, 77.2]	<0.001
Male sex, *n* (%)	1,075 (48.2)	195 (49.5)	242 (61.6)	<0.001
Race, *n* (%)				<0.001
Other	634 (28.4)	144 (36.5)	171 (43.5)	
White	1,341 (60.2)	208 (52.8)	170 (43.3)	
Black	254 (11.4)	42 (10.7)	52 (13.2)	
Comorbidities				
Charlson comorbidity index	4.00 [3.00, 5.00]	4.00 [3.00, 5.00]	4.00 [3.00, 5.00]	0.339
Hypertension, *n* (%)	1,123 (50.4)	203 (51.5)	202 (51.4)	0.873
Coronary heart disease, *n* (%)	549 (24.6)	85 (21.6)	103 (26.2)	0.292
Atrial fibrillation, *n* (%)	888 (39.8)	153 (38.8)	145 (36.9)	0.533
Chronic pulmonary disease, *n* (%)	376 (16.9)	72 (18.3)	65 (16.5)	0.764
Myocardial infarction, *n* (%)	360 (16.2)	75 (19.0)	74 (18.8)	0.200
Congestive heart failure, *n* (%)	606 (27.2)	98 (24.9)	112 (28.5)	0.500
Diabetes without complications, *n* (%)	553 (24.8)	117 (29.7)	127 (32.3)	0.002
Diabetes with complications, *n* (%)	275 (12.3)	54 (13.7)	46 (11.7)	0.672
Renal disease, *n* (%)	440 (19.7)	92 (23.4)	77 (19.6)	0.245
Mild liver disease, *n* (%)	118 (5.29)	20 (5.08)	30 (7.63)	0.158
Illness severity, infection and organ support				
SOFA score	3.00 [1.00, 5.00]	4.00 [2.00, 6.00]	5.00 [3.00, 7.00]	<0.001
SAPS II score	31.0 [24.0, 40.0]	35.0 [29.0, 43.0]	36.0 [29.0, 45.0]	<0.001
Sepsis, *n* (%)	637 (28.6)	236 (59.9)	315 (80.2)	<0.001
Mechanical ventilation, *n* (%)	576 (25.8)	229 (58.1)	298 (75.8)	<0.001
Haemopurification or RRT, *n* (%)	110 (4.93)	36 (9.14)	31 (7.89)	0.001
Laboratory measurements				
White blood cell count, ×10^9^/L	9.30 [7.20, 12.2]	11.1 [8.30, 14.1]	11.2 [8.20, 14.7]	<0.001
Hemoglobin, g/dL	12.3 [10.6, 13.7]	11.8 [10.0, 13.3]	11.6 [10.0, 13.3]	<0.001
Platelet count, ×10^9^/L	212 [168, 266]	209 [160, 275]	203 [151, 264]	0.033
Serum creatinine, mg/dL	0.90 [0.70, 1.20]	0.90 [0.70, 1.40]	1.00 [0.80, 1.37]	0.003
Blood urea nitrogen, mg/dL	17.0 [13.0, 25.0]	18.0 [13.0, 27.0]	18.0 [13.0, 27.0]	0.050
Glucose, mg/dL	119 [99.0, 152]	130 [109, 175]	138 [114, 181]	<0.001
International normalized ratio	1.20 [1.10, 1.30]	1.20 [1.10, 1.30]	1.20 [1.10, 1.40]	0.001
Early ICU physiological variables				
Heart rate, beats/min	77.4 [68.8, 88.3]	83.5 [71.9, 95.0]	85.0 [75.0, 97.1]	<0.001
Mean arterial pressure, mmHg	88.1 [79.5, 98.0]	84.9 [76.3, 93.5]	84.5 [75.5, 93.8]	<0.001
Respiratory rate, breaths/min	18.4 [16.7, 20.5]	19.2 [17.3, 21.5]	20.1 [17.9, 23.1]	<0.001
SpO_2_, %	96.8 [95.6, 98.0]	97.5 [96.1, 98.7]	97.9 [96.6, 99.1]	<0.001
Mean body temperature, °C	36.8 [36.7, 37.0]	37.1 [36.9, 37.4]	37.4 [37.1, 37.8]	<0.001
Temperature exposure and monitoring during the first 72 h				
Number of temperature measurements	16.0 [10.0, 18.0]	19.0 [16.0, 20.0]	20.0 [17.0, 23.0]	<0.001
Observed temperature-monitoring time, h	60.7 [36.0, 68.2]	68.0 [60.8, 69.7]	68.0 [60.4, 69.9]	<0.001
Temperature measurement density, measurements/24 h	6.51 [6.19, 6.94]	6.76 [6.42, 7.27]	7.58 [6.86, 8.79]	<0.001
Maximum temperature, °C	37.3 [37.1, 37.6]	38.2 [38.0, 38.3]	38.9 [38.6, 39.3]	<0.001
Cumulative fever burden, °C-hours	0.00 [0.00, 0.00]	0.40 [0.10, 0.90]	4.90 [2.70, 10.1]	<0.001
Daily mean fever burden, °C-hours/24 h	0.00 [0.00, 0.00]	0.20 [0.00, 0.40]	2.00 [1.10, 4.10]	<0.001
Clinical outcomes				
ICU length of stay, days	3.06 [1.83, 5.79]	5.82 [3.15, 9.80]	7.73 [4.33, 13.7]	<0.001
Hospital length of stay, days	7.75 [4.06, 14.5]	12.8 [7.10, 21.9]	14.9 [8.14, 25.8]	<0.001
28-day all-cause mortality, *n* (%)	340 (15.3)	113 (28.7)	144 (36.6)	<0.001

Values are median [interquartile range] or *n* (%). *p* values were derived from the Kruskal-Wallis test, chi-square test, or Fisher exact test, as appropriate. Fever burden was calculated during the first 72 h after ICU admission and standardized to 24 h. The primary outcome was 28-day all-cause mortality from ICU admission.

ICU, intensive care unit; INR, international normalized ratio; IQR, interquartile range; RRT, renal replacement therapy; SAPS II, Simplified Acute Physiology Score II; SOFA, Sequential Organ Failure Assessment; SpO_2_, peripheral oxygen saturation; WBC, white blood cell.

### Unadjusted survival analysis

**Figure 2:**
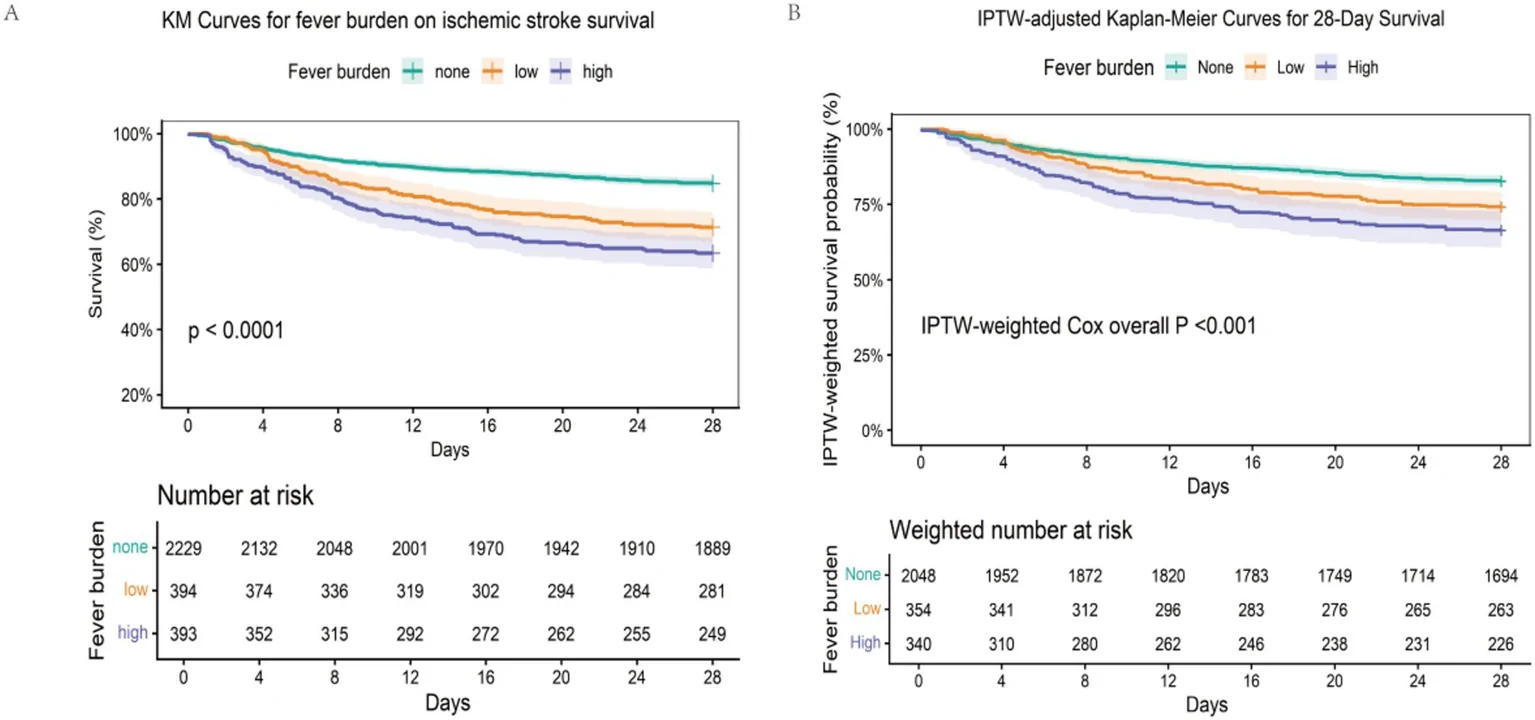
Kaplan-Meier curves for 28-day survival according to fever-burden group. **(A)** Unadjusted Kaplan-Meier curves. **(B)** IPTW-adjusted Kaplan-Meier curves. IPTW, inverse probability of treatment weighting.

Unadjusted Kaplan–Meier curves (Figure 2A) showed lower 28-day survival in patients with fever burden during the first 72 h after ICU admission. The estimated 28-day survival was 84.7% in the no fever-burden group, 71.3% in the low fever-burden group, and 63.4% in the high fever-burden group. The corresponding 28-day mortality rates were 15.3, 28.7, and 36.6%, respectively. Survival distributions differed significantly across the three groups by the log-rank test (*p* < 0.001).

### Association between fever burden and 28-day mortality

In the multiply imputed Cox models (Table 2), higher early ICU fever burden was associated with an increased hazard of 28-day all-cause mortality. In the crude model, compared with the no fever-burden group, the low and high fever-burden groups had higher hazards of death, with HRs of 2.018 (95% CI 1.630–2.498; *p* < 0.001) and 2.761 (95% CI 2.271–3.356; *p* < 0.001), respectively. After full adjustment, the associations were attenuated but remained statistically significant. In Model 3, the adjusted HR was 1.404 (95% CI 1.126–1.751; *p* = 0.003) for the low fever-burden group and 1.948 (95% CI 1.564–2.427; *p* < 0.001) for the high fever-burden group. Univariable Cox regression results are provided in Supplementary Table 3.

**Table 2:** Association between 72-h fever-burden group and 28-day all-cause mortality in multiply imputed Cox models.

Model	Fever-burden group	HR (95% CI)	*p* value
Crude model	Low fever burden	2.018 (1.630–2.498)	<0.001
	High fever burden	2.761 (2.271–3.356)	<0.001
Model 1	Low fever burden	1.721 (1.384–2.139)	<0.001
	High fever burden	2.479 (1.998–3.076)	<0.001
Model 2	Low fever burden	1.604 (1.289–1.995)	<0.001
	High fever burden	2.246 (1.806–2.793)	<0.001
Model 3	Low fever burden	1.404 (1.126–1.751)	0.003
	High fever burden	1.948 (1.564–2.427)	<0.001

Values are hazard ratios with 95% confidence intervals. The no fever-burden group was used as the reference. Cox models were fitted across 20 multiply imputed datasets and pooled using Rubin’s rules.

The crude model included fever-burden group only.

Model 1 adjusted for age, sex, race, diabetes mellitus, coronary heart disease, atrial fibrillation and hypertension. Model 2 further adjusted for SOFA score, heart rate, respiratory rate, SpO_2_, white blood cell count and INR. Model 3 additionally adjusted for mechanical ventilation and haemopurification or renal replacement therapy.

CI, confidence interval; HR, hazard ratio; INR, international normalized ratio; SOFA, Sequential Organ Failure Assessment; SpO_2_, peripheral oxygen saturation.

### Sensitivity analyses

The association between early ICU fever burden and 28-day all-cause mortality was generally robust across prespecified sensitivity analyses. In complete-case Cox models, both low and high 72-h fever-burden groups remained associated with higher mortality after full adjustment compared with the no fever-burden group (low: HR 1.388, 95% CI 1.103–1.747; high: HR 1.925, 95% CI 1.531–2.419). Similar estimates were observed after additional adjustment for temperature measurement density (low: HR 1.404, 95% CI 1.115–1.768; high: HR 1.890, 95% CI 1.503–2.376).

Analyses using alternative exposure windows showed a comparable pattern for the 48-h fever-burden window, in which both low and high fever burden remained associated with higher 28-day mortality after full adjustment (low: HR 1.504, 95% CI 1.183–1.912; high: HR 1.620, 95% CI 1.276–2.055). For the 24-h window, low fever burden remained associated with higher mortality (HR 1.418, 95% CI 1.080–1.862), whereas the association for high fever burden was attenuated and no longer statistically significant (HR 1.209, 95% CI 0.918–1.594). The day-3 landmark analysis also supported the association of both low and high 72-h fever burden with 28-day mortality (low: HR 1.562, 95% CI 1.219–2.002; high: HR 1.943, 95% CI 1.502–2.513). The findings were further supported by analyses using alternative temperature thresholds and by analyses excluding patients with sepsis. In the supplementary day-7 landmark analysis, 7-day fever burden was also associated with subsequent 28-day mortality. Detailed results are shown in Table 3 and Supplementary Tables 4–11.

**Table 3:** Summary of sensitivity analyses for the association between fever burden and 28-day all-cause mortality.

Sensitivity analysis	Comparison	HR (95% CI)	*p* value
Complete-case Cox analysis	Low fever burden	1.388 (1.103–1.747)	0.005
	High fever burden	1.925 (1.531–2.419)	<0.001
Additional adjustment for temperature measurement density	Low fever burden	1.404 (1.115–1.768)	0.004
	High fever burden	1.890 (1.503–2.376)	<0.001
24-h fever-burden window	Low fever burden	1.418 (1.080–1.862)	0.012
	High fever burden	1.209 (0.918–1.594)	0.177
48-h fever-burden window	Low fever burden	1.504 (1.183–1.912)	<0.001
	High fever burden	1.620 (1.276–2.055)	<0.001
Day-3 landmark analysis	Low fever burden	1.562 (1.219–2.002)	<0.001
	High fever burden	1.943 (1.502–2.513)	<0.001
Alternative threshold: 37.5 °C	Low fever burden	1.285 (1.026–1.611)	0.029
	High fever burden	1.831 (1.475–2.273)	<0.001
Alternative threshold: 37.8 °C	Low fever burden	1.569 (1.256–1.959)	<0.001
	High fever burden	1.948 (1.557–2.436)	<0.001
Alternative threshold: 38.0 °C	Low fever burden	1.352 (1.063–1.720)	0.014
	High fever burden	1.827 (1.446–2.310)	<0.001
Alternative threshold: 38.3°C	Low fever burden	1.548 (1.197–2.002)	<0.001
	High fever burden	2.009 (1.556–2.595)	<0.001
Excluding patients with sepsis	Low fever burden	1.628 (1.103–2.402)	0.014
	High fever burden	3.571 (2.292–5.565)	<0.001
Seven-day fever-burden landmark analysis	Low fever burden	1.724 (1.283–2.317)	<0.001
	High fever burden	2.372 (1.744–3.227)	<0.001

Values are hazard ratios with 95% confidence intervals from fully adjusted Cox models unless otherwise stated. The no fever-burden group was the reference. Landmark analyses excluded patients who died or were censored before the landmark time.

CI, confidence interval; HR, hazard ratio.

### IPTW analysis

**Figure 3:**
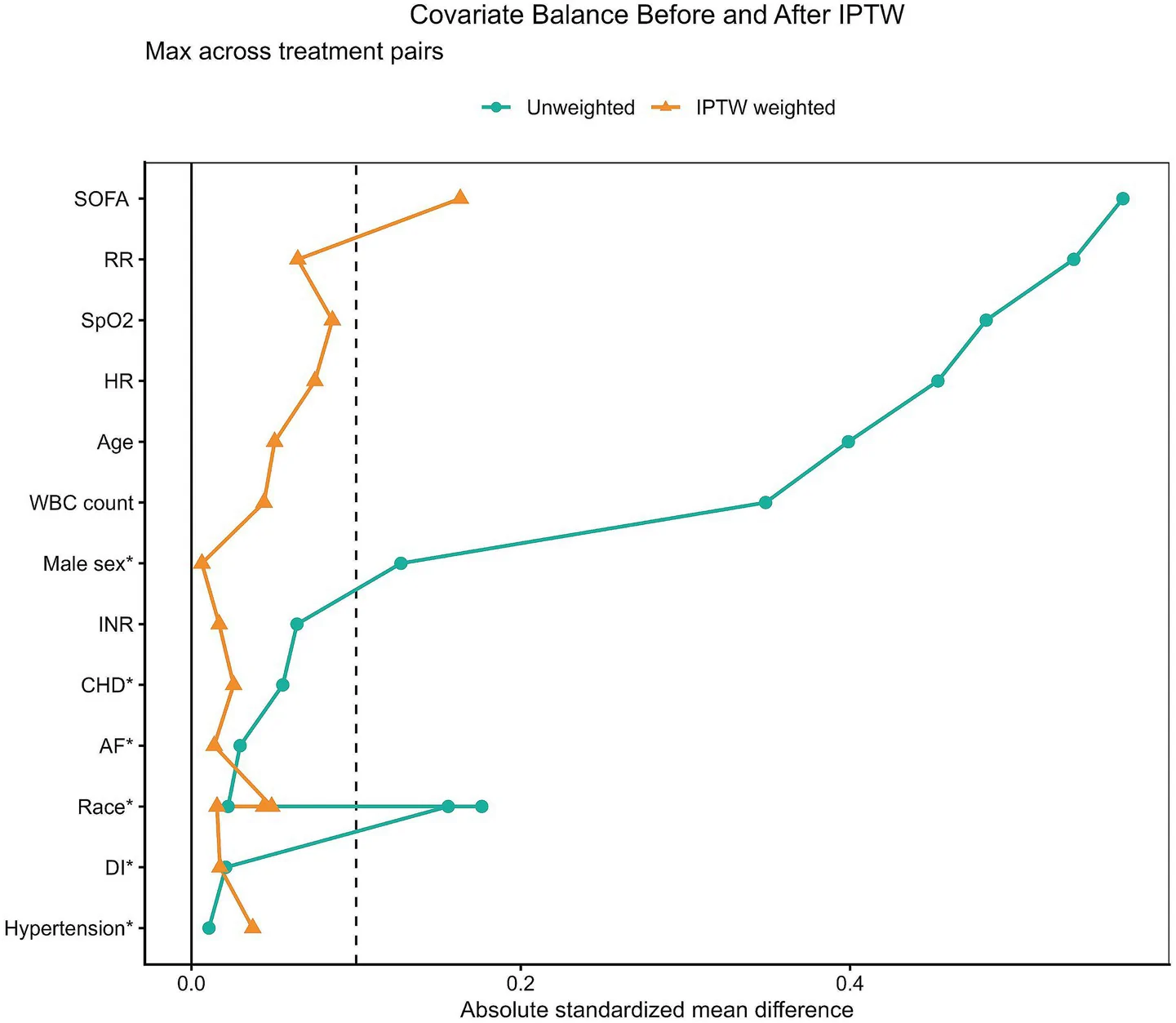
Covariate balance before and after IPTW.

After excluding patients with missing covariates required for the propensity-score model, the IPTW analysis included 2,781 patients, of whom 544 died within 28 days. Stabilized inverse probability weights were estimated using a multinomial propensity-score model and trimmed at the 1st and 99th percentiles. The mean stabilized weight was 0.986, and the median weight was 0.898. After weighting, covariate balance improved substantially for most measured covariates. However, some residual imbalance remained for the SOFA score, with the maximum pairwise absolute standardized mean difference remaining above 0.10. After weighting, covariate balance was assessed using standardized mean differences and is shown in Figure 3.

In the IPTW-weighted Cox model (Table 4), both low and high fever burden remained associated with higher 28-day all-cause mortality compared with no fever burden. The HR was 1.553 (95% CI 1.223–1.973; *p* < 0.001) for low fever burden and 2.167 (95% CI 1.687–2.783; *p* < 0.001) for high fever burden, with an overall *p* value <0.001. The IPTW-adjusted Kaplan–Meier curves (Figure 2B), displayed together with the unadjusted curves, showed a similar graded separation across fever-burden groups, with the lowest 28-day survival in the high fever-burden group.

**Table 4:** IPTW-weighted Cox regression for the association between 72-h fever-burden group and 28-day all-cause mortality.

Fever-burden group	HR (95% CI)	*p* value
No fever burden	Reference	
Low fever burden	1.553 (1.223–1.973)	<0.001
High fever burden	2.167 (1.687–2.783)	<0.001

Values are hazard ratios with 95% confidence intervals from IPTW-weighted Cox regression. Stabilized inverse probability weights were estimated using a multinomial propensity-score model and trimmed at the 1st and 99th percentiles. The no fever-burden group was the reference. Overall *p* < 0.001.

CI, confidence interval; HR, hazard ratio; IPTW, inverse probability of treatment weighting.

### Subgroup analyses

**Figure 4:**
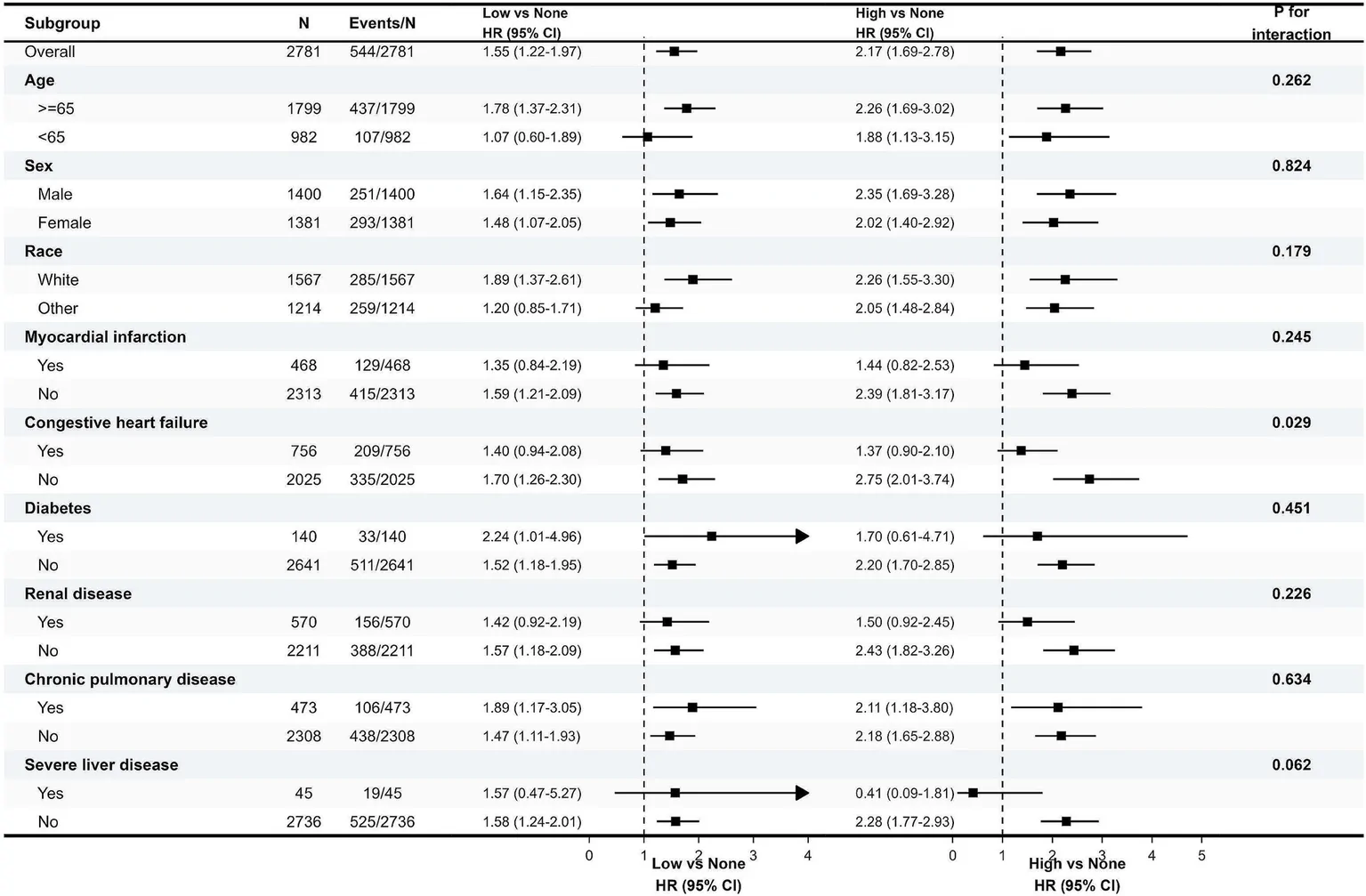
Subgroup analyses and interaction tests.

Subgroup analyses were performed across predefined patient strata. The association between fever burden and 28-day all-cause mortality showed a generally similar direction across most subgroups. High fever burden tended to be associated with higher mortality in most strata, although the precision of estimates varied across subgroups. No statistically significant interactions were observed for most subgroup variables. An interaction was observed for congestive heart failure (*p* for interaction = 0.029), suggesting possible heterogeneity in the association according to heart failure status. Given the exploratory nature of subgroup analyses, this finding should be interpreted cautiously. The subgroup results are shown in Figure 4.

## Discussion

In this retrospective cohort of ICU patients with acute ischaemic stroke, greater fever burden during the first 72 h after ICU admission was associated with higher 28-day all-cause mortality. This association remained evident after adjustment for measured covariates and was broadly consistent across the IPTW and sensitivity analyses. These findings suggest that a time-integrated measure of cumulative thermal exposure may provide prognostic information beyond isolated temperature measurements during the early ICU period, although its clinical utility requires further prospective validation.

Compared with maximum temperature or a binary definition of fever, fever burden incorporates both the magnitude and duration of temperature elevation, and may therefore better reflect cumulative thermal exposure during the early ICU period (12, 13). This distinction is clinically relevant in acute ischaemic stroke, because ischaemic brain tissue and the surrounding penumbra may be vulnerable to sustained temperature elevation (15, 16). Elevated temperature can increase cerebral metabolic demand and oxygen consumption, and may aggravate secondary injury through inflammatory activation, blood–brain barrier disruption and cerebral oedema (5, 17, 18). However, fever burden in critically ill patients with acute ischaemic stroke should not be interpreted as a purely neurological phenomenon. In the ICU setting, early fever may arise from multiple overlapping processes, including infection or sepsis, stroke-related thermoregulatory dysfunction, systemic inflammation, stroke-induced immunosuppression and overall illness severity (4, 7, 19, 20). Therefore, the observed association between fever burden and mortality may reflect both cumulative thermal stress and the broader physiological burden of critical illness. This interpretation is supported by the sensitivity analysis excluding patients with sepsis, while also highlighting the possibility of residual infection-related and stroke-severity confounding.

Previous studies on temperature abnormalities in acute ischaemic stroke have commonly relied on admission temperature, early temperature changes, maximum temperature, or binary fever definitions (21–23). Although these measures are clinically useful, they do not fully capture the persistence and cumulative magnitude of thermal exposure (12). A recent systematic review and meta-analysis of non-anoxic acute brain injury also reported that fever was associated with poor neurological outcomes and mortality (18). In the INTREPID randomized clinical trial, fever prevention reduced fever burden in patients with acute vascular brain injury but did not significantly improve 3-month functional outcomes (13). Similarly, evidence from broader critically ill populations suggests that antipyretic or temperature-control strategies can reduce temperature but have not consistently improved survival or other clinical outcomes (24). Together, these findings suggest that fever burden may be useful as a prognostic marker, while its role as a modifiable treatment target remains uncertain. Accordingly, by examining fever exposure from a cumulative perspective, the present study adds to the evidence that early fever burden may provide prognostic information in critically ill patients with acute ischaemic stroke.

In subgroup analyses, a significant interaction was observed for congestive heart failure, suggesting possible heterogeneity in the association between fever burden and 28-day mortality according to heart failure status. This finding may reflect differences in cardiovascular reserve, haemodynamic vulnerability and competing mortality risks among patients with pre-existing heart failure. Given the exploratory nature of subgroup analyses and the possibility of multiple testing, this result should be interpreted cautiously.

Several limitations should be acknowledged. First, this retrospective single-centre study was based on a single critical care database, which may introduce selection bias, information bias and limited generalisability. Second, several stroke-specific severity and treatment variables were not consistently available, including NIHSS score, infarct volume, vascular territory, large-vessel occlusion, reperfusion therapy, haemorrhagic transformation, cerebral oedema and decompressive surgery. Residual confounding by stroke severity, infarct characteristics and treatment strategy may therefore persist despite multivariable adjustment, multiple imputation and IPTW analysis. In addition, the SOFA score retained some residual imbalance after IPTW, suggesting that confounding by overall illness severity may not have been completely eliminated. Third, the etiology of fever could not be reliably determined, and central fever could not be clearly distinguished from infection-related fever. Although white blood cell count was included in the adjusted model and a sensitivity analysis excluding patients with sepsis was performed, residual infection-related confounding and fever-etiology misclassification cannot be excluded. Fourth, although we adjusted for temperature measurement density, residual bias related to temperature monitoring practices and antipyretic or cooling interventions may remain. Therefore, the findings should be interpreted as associations after adjustment for measured covariates, rather than evidence of a causal effect of fever burden on mortality. Future multicentre prospective studies with standardized temperature monitoring, detailed stroke-severity assessment, infection adjudication, treatment information and temperature-management data are needed to validate these findings and clarify whether fever burden can improve risk stratification or guide temperature-management strategies in selected patients with acute ischaemic stroke (17).

## Conclusion

In this retrospective cohort of ICU patients with acute ischaemic stroke, greater fever burden during the first 72 h after ICU admission was associated with higher 28-day all-cause mortality. This association was generally consistent across sensitivity, IPTW and subgroup analyses. Early ICU fever burden may serve as a time-integrated prognostic marker, but its value as a target for temperature-management strategies requires further prospective validation.

## Data Availability

The data analyzed in this study are available from the MIMIC-IV database via PhysioNet. Access to the database requires completion of the required training and approval of the data use agreement. The authors are not authorized to redistribute the original database or provide individual-level raw data.

## Glossary

AIS: acute ischemic stroke
ASMD: absolute standardized mean difference
ATE: average treatment effect
AUC: area under the curve
BIDMC: Beth Israel Deaconess Medical Center
CCI: Charlson Comorbidity Index
CI: confidence interval
COPD: chronic obstructive pulmonary disease
DBP: diastolic blood pressure
EHR: electronic health record
HIPAA: Health Insurance Portability and Accountability Act
HR: hazard ratio
ICD-10: International Classification of Diseases, Tenth Revision
ICD-9: International Classification of Diseases, Ninth Revision
ICU: intensive care unit
INR: international normalized ratio
IPTW: inverse probability of treatment weighting
IQR: interquartile range
IRB: Institutional Review Board
KM: Kaplan–Meier
LOS: length of stay
MBP: mean blood pressure
MIMIC-IV: Medical Information Mart for Intensive Care IV
MIT: Massachusetts Institute of Technology
RR: respiratory rate
RRT: renal replacement therapy
SAP: stroke-associated pneumonia
SAPS II: Simplified Acute Physiology Score II
SBP: systolic blood pressure
SD: standard deviation
SOFA: Sequential Organ Failure Assessment
SpO2: peripheral oxygen saturation
Tmax: maximum temperature
VIF: variance inflation factor
WBC: white blood cell count
