# Supplementary material for: Association between early ICU fever burden and 28-day mortality in patients with acute ischemic stroke: a retrospective cohort study based on the MIMIC-IV database
Source: Front Neurol. 2026 Aug 20;17:1900723. doi: 10.3389/fneur.2026.1900723 (PMC13537944; doi:10.3389/fneur.2026.1900723)
Supplement: Supplementary file 1 [file Data_Sheet_1.docx]

**Supplementary Material Figure 1** Directed acyclic graph

^[1]^Directed acyclic graph for the association between early ICU fever burden and 28-day all-cause mortality. Solid boxes indicate measured variables addressed in the primary, extended or sensitivity analyses. Dashed boxes indicate unmeasured factors.

***Abbreviations****：ICU, intensive care unit.*

**Supplementary Material Figure 2** Convergence diagnostics for multiple imputation by chained equations

**Supplementary Material Table 1** Missing data for variables in the analytical dataset

| **Domain** | **Variable** | **Missing n (%)** |
| --- | --- | --- |
| Vital signs | Heart rate, mean | 1 (0.0%) |
| Vital signs | Systolic blood pressure, mean | 1 (0.0%) |
| Vital signs | Diastolic blood pressure, mean | 1 (0.0%) |
| Vital signs | Mean arterial pressure, mean | 1 (0.0%) |
| Vital signs | Peripheral oxygen saturation, mean | 1 (0.0%) |
| Vital signs | Peripheral oxygen saturation, maximum | 1 (0.0%) |
| Vital signs | Respiratory rate, mean | 9 (0.3%) |
| Vital signs | Respiratory rate, maximum | 9 (0.3%) |
| Laboratory tests | Glucose, mean | 26 (0.9%) |
| Laboratory tests | Glucose, maximum | 26 (0.9%) |
| Laboratory tests | Haematocrit, first value | 29 (1.0%) |
| Temperature exposure and monitoring | Temperature measurements within 48 h | 29 (1.0%) |
| Temperature exposure and monitoring | Observed temperature-monitoring hours within 48 h | 29 (1.0%) |
| Temperature exposure and monitoring | Cumulative fever burden within 48 h | 29 (1.0%) |
| Temperature exposure and monitoring | Mean temperature within 48 h | 29 (1.0%) |
| Temperature exposure and monitoring | Maximum temperature within 48 h | 29 (1.0%) |
| Temperature exposure and monitoring | Time-normalised fever burden within 48 h | 29 (1.0%) |
| Vital signs | Body temperature, mean | 38 (1.3%) |
| Vital signs | Body temperature, maximum | 38 (1.3%) |
| Temperature exposure and monitoring | Fever-burden group within 48 h | 43 (1.4%) |
| Laboratory tests | Platelet count, first value | 48 (1.6%) |
| Laboratory tests | Haemoglobin, first value | 49 (1.6%) |
| Laboratory tests | White blood cell count, first value | 50 (1.7%) |
| Laboratory tests | Blood urea nitrogen, first value | 95 (3.1%) |
| Temperature exposure and monitoring | Temperature measurements within 24 h | 98 (3.2%) |
| Temperature exposure and monitoring | Observed temperature-monitoring hours within 24 h | 98 (3.2%) |
| Temperature exposure and monitoring | Cumulative fever burden within 24 h | 98 (3.2%) |
| Temperature exposure and monitoring | Mean temperature within 24 h | 98 (3.2%) |
| Temperature exposure and monitoring | Maximum temperature within 24 h | 98 (3.2%) |
| Temperature exposure and monitoring | Time-normalised fever burden within 24 h | 98 (3.2%) |
| Temperature exposure and monitoring | Fever-burden group within 24 h | 125 (4.1%) |
| Laboratory tests | Serum creatinine, first value | 138 (4.6%) |
| Laboratory tests | Chloride, first value | 168 (5.6%) |
| Laboratory tests | Activated partial thromboplastin time, first value | 168 (5.6%) |
| Laboratory tests | Bicarbonate, first value | 178 (5.9%) |
| Laboratory tests | Sodium, first value | 182 (6.0%) |
| Laboratory tests | Potassium, first value | 185 (6.1%) |
| Laboratory tests | Anion gap, first value | 186 (6.2%) |
| Laboratory tests | Prothrombin time, first value | 200 (6.6%) |
| Laboratory tests | International normalised ratio, first value | 201 (6.7%) |
| Laboratory tests | Glucose, first value | 222 (7.4%) |
| Laboratory tests | Calcium, first value | 555 (18.4%) |
| Blood gas variables | pH | 1723 (57.1%) |
| Blood gas variables | Partial pressure of oxygen | 1723 (57.1%) |
| Blood gas variables | Partial pressure of carbon dioxide | 1723 (57.1%) |
| Blood gas variables | Base excess | 1724 (57.2%) |
| Outcome source variables | Date of death | 1892 (62.7%) |
| Outcome source variables | Survival time, days | 1892 (62.7%) |
| Differential blood count | Absolute basophil count, first value | 2093 (69.4%) |
| Differential blood count | Absolute eosinophil count, first value | 2093 (69.4%) |
| Differential blood count | Absolute lymphocyte count, first value | 2093 (69.4%) |
| Differential blood count | Absolute monocyte count, first value | 2093 (69.4%) |
| Differential blood count | Absolute neutrophil count, first value | 2093 (69.4%) |
| Differential blood count | Basophils, first value | 2093 (69.4%) |
| Differential blood count | Eosinophils, first value | 2093 (69.4%) |
| Differential blood count | Lymphocytes, first value | 2093 (69.4%) |
| Differential blood count | Monocytes, first value | 2093 (69.4%) |
| Differential blood count | Neutrophils, first value | 2093 (69.4%) |
| Laboratory tests | Lactate | 2121 (70.3%) |
| Laboratory tests | Immature granulocytes, first value | 2340 (77.6%) |
| Laboratory tests | Band neutrophils, first value | 2914 (96.6%) |
| Laboratory tests | Metamyelocytes, first value | 2926 (97.0%) |
| Laboratory tests | Atypical lymphocytes, first value | 2932 (97.2%) |
| Laboratory tests | Nucleated red blood cells, first value | 2956 (98.0%) |
| Laboratory tests | Bicarbonate | 2984 (98.9%) |

^[2]^Values are n (%). Percentages were calculated using the final analytical cohort as the denominator (N = 3016).Variables with >50% missingness were excluded.

**Supplementary Material Table 2** Proportional hazards assumption tests for complete-case Cox models

| **Model** | **Variable** | **Chi-square** | **df** | **P value** | **PH assumption** |
| --- | --- | --- | --- | --- | --- |
| Crude model | Fever-burden group | 1.923 | 2 | 0.382 | Met |
| Crude model | GLOBAL | 1.923 | 2 | 0.382 | Met |
| Model 1 | Fever-burden group | 2.559 | 2 | 0.278 | Met |
| Model 1 | Age | 1.167 | 1 | 0.280 | Met |
| Model 1 | Sex | 1.549 | 1 | 0.213 | Met |
| Model 1 | Heart rate | 2.020 | 1 | 0.155 | Met |
| Model 1 | Respiratory rate | 0.665 | 1 | 0.415 | Met |
| Model 1 | SpO₂ | 0.601 | 1 | 0.438 | Met |
| Model 1 | Coronary heart disease | 1.764 | 1 | 0.184 | Met |
| Model 1 | Atrial fibrillation | 0.145 | 1 | 0.704 | Met |
| Model 1 | Hypertension | 2.765 | 1 | 0.096 | Met |
| Model 1 | GLOBAL | 14.988 | 10 | 0.132 | Met |
| Model 2 | Fever-burden group | 2.169 | 2 | 0.338 | Met |
| Model 2 | Age | 1.082 | 1 | 0.298 | Met |
| Model 2 | Sex | 1.182 | 1 | 0.277 | Met |
| Model 2 | SOFA score | 0.080 | 1 | 0.777 | Met |
| Model 2 | Heart rate | 2.711 | 1 | 0.100 | Met |
| Model 2 | Respiratory rate | 0.259 | 1 | 0.611 | Met |
| Model 2 | SpO₂ | 0.883 | 1 | 0.347 | Met |
| Model 2 | White blood cell count | 0.282 | 1 | 0.596 | Met |
| Model 2 | INR | 1.809 | 1 | 0.179 | Met |
| Model 2 | Coronary heart disease | 1.647 | 1 | 0.199 | Met |
| Model 2 | Atrial fibrillation | 0.138 | 1 | 0.710 | Met |
| Model 2 | Hypertension | 2.917 | 1 | 0.088 | Met |
| Model 2 | GLOBAL | 15.037 | 13 | 0.305 | Met |
| Model 3 | Fever-burden group | 1.600 | 2 | 0.449 | Met |
| Model 3 | Age | 0.968 | 1 | 0.325 | Met |
| Model 3 | Sex | 1.214 | 1 | 0.270 | Met |
| Model 3 | SOFA score | 0.000 | 1 | 0.994 | Met |
| Model 3 | Heart rate | 2.543 | 1 | 0.111 | Met |
| Model 3 | Respiratory rate | 0.571 | 1 | 0.450 | Met |
| Model 3 | SpO₂ | 0.448 | 1 | 0.503 | Met |
| Model 3 | White blood cell count | 0.182 | 1 | 0.670 | Met |
| Model 3 | INR | 1.570 | 1 | 0.210 | Met |
| Model 3 | Coronary heart disease | 1.723 | 1 | 0.189 | Met |
| Model 3 | Atrial fibrillation | 0.137 | 1 | 0.711 | Met |
| Model 3 | Hypertension | 3.161 | 1 | 0.075 | Met |
| Model 3 | Mechanical ventilation | 0.195 | 1 | 0.659 | Met |
| Model 3 | Haemopurification or RRT | 5.786 | 1 | 0.016 | Potential violation |
| Model 3 | GLOBAL | 18.698 | 15 | 0.228 | Met |

^[3]^The proportional hazards assumption was assessed using Schoenfeld residuals in complete-case Cox models. A P value <0.05 suggests a potential violation of the proportional hazards assumption. GLOBAL indicates the overall test for each model. The fever-burden group satisfied the proportional hazards assumption in all models, and global tests were not significant across models.

***Abbreviations****：INR, international normalised ratio; PH, proportional hazards; RRT, renal replacement therapy; SOFA, Sequential Organ Failure Assessment; SpO₂, peripheral oxygen saturation.*

**Supplementary Material Table 3** Univariable Cox regression analysis for 28-day all-cause mortality

| **Variable** | **HR (95% CI)** | **P value** |
| --- | --- | --- |
| Fever burden | 2.007 (1.607-2.506) | <0.001 |
| Fever burden | 2.675 (2.182-3.281) | <0.001 |
| Sex | 0.824 (0.696-0.975) | 0.024 |
| Age | 1.038 (1.031-1.045) | <0.001 |
| Race | 0.773 (0.646-0.926) | 0.005 |
| Race | 0.743 (0.554-0.997) | 0.048 |
| Myocardial infarction | 1.601 (1.314-1.951) | <0.001 |
| Congestive heart failure | 1.762 (1.482-2.094) | <0.001 |
| Chronic pulmonary disease | 1.203 (0.973-1.488) | 0.087 |
| Diabetes with complications | 1.126 (0.884-1.432) | 0.336 |
| Renal disease | 1.628 (1.352-1.960) | <0.001 |
| Severe liver disease | 2.439 (1.543-3.855) | <0.001 |
| Heart rate | 1.022 (1.017-1.027) | <0.001 |
| Systolic blood pressure | 0.996 (0.992-1.001) | 0.087 |
| Diastolic blood pressure | 0.978 (0.972-0.985) | <0.001 |
| Respiratory rate | 1.098 (1.076-1.122) | <0.001 |
| Maximum temperature during 72 h | 1.737 (1.562-1.931) | <0.001 |
| SpO2 | 1.149 (1.092-1.208) | <0.001 |
| Haemoglobin | 0.892 (0.862-0.924) | <0.001 |
| White blood cell count | 1.048 (1.038-1.058) | <0.001 |
| Creatinine | 1.110 (1.067-1.154) | <0.001 |
| Sodium | 1.003 (0.983-1.023) | 0.789 |
| Potassium | 1.275 (1.152-1.412) | <0.001 |
| Anion gap | 1.072 (1.055-1.089) | <0.001 |
| INR | 1.180 (1.111-1.253) | <0.001 |
| SOFA score | 1.154 (1.130-1.178) | <0.001 |
| Mechanical ventilation | 3.144 (2.643-3.741) | <0.001 |
| Haemopurification | 2.277 (1.754-2.955) | <0.001 |

^[4]^Univariable Cox proportional hazards models were fitted in the complete-case cohort using 28-day follow-up time and 28-day all-cause mortality status. The no fever-burden group was used as the reference for fever-burden comparisons. For binary variables, the reference category was absence of the condition or female sex, as appropriate. For race, other race was used as the reference. Hazard ratios for continuous variables are expressed per one-unit increase in the unit used in the analysis dataset.

***Abbreviations****：CI, confidence interval; HR, hazard ratio; INR, international normalised ratio; SOFA, Sequential Organ Failure Assessment; SpO2, peripheral oxygen saturation.*

**Supplementary Material Table 4** Temperature measurement frequency by fever-burden group

| **Fever-burden group** | **N** | **Measurements, median [IQR]** | **Observed hours, median [IQR]** | **Measurements per 24 h, median [IQR]** |
| --- | --- | --- | --- | --- |
| No fever burden | 2,039 | 16 [10-18] | 61.1 [36.0-68.3] | 6.51 [6.20-6.94] |
| Low fever burden | 368 | 19 [16-20] | 68.0 [62.5-69.7] | 6.75 [6.42-7.27] |
| High fever burden | 374 | 20 [17-23] | 68.0 [60.4-69.9] | 7.56 [6.86-8.80] |

^[5]^Values are median [interquartile range] unless otherwise indicated. Temperature measurement density was calculated as the number of temperature measurements divided by observed hours and standardized to measurements per 24 hours.

**Supplementary Material Table 5** Complete-case Cox sensitivity analysis

| **Model** | **Group** | **HR (95% CI)** | **P value** |
| --- | --- | --- | --- |
| Crude model | Low fever burden | 2.007 (1.607-2.506) | <0.001 |
| Crude model | High fever burden | 2.675 (2.182-3.281) | <0.001 |
| Model 1 | Low fever burden | 1.698 (1.353-2.130) | <0.001 |
| Model 1 | High fever burden | 2.405 (1.920-3.012) | <0.001 |
| Model 2 | Low fever burden | 1.584 (1.261-1.989) | <0.001 |
| Model 2 | High fever burden | 2.180 (1.736-2.737) | <0.001 |
| Model 3 | Low fever burden | 1.388 (1.103-1.747) | 0.005 |
| Model 3 | High fever burden | 1.925 (1.531-2.419) | <0.001 |

*^[6]^* Complete-case Cox models were performed as a sensitivity analysis.

The no fever-burden group was used as the reference.

Model 1 adjusted for age, sex, race, diabetes mellitus, coronary heart disease, atrial fibrillation and hypertension.

Model 2 further adjusted for SOFA score, heart rate, respiratory rate, SpO2, white blood cell count and INR.

Model 3 additionally adjusted for mechanical ventilation and haemopurification or renal replacement therapy.

**Supplementary Material Table 6** Sensitivity analysis additionally adjusted for temperature measurement density

| **Model** | **Group** | **HR (95% CI)** | **P value** |
| --- | --- | --- | --- |
| Crude model | Low fever burden | 2.007 (1.607-2.506) | <0.001 |
| Crude model | High fever burden | 2.675 (2.182-3.281) | <0.001 |
| Fully adjusted model additionally adjusted for measurement density | Low fever burden | 1.404 (1.115-1.768) | 0.004 |
| Fully adjusted model additionally adjusted for measurement density | High fever burden | 1.890 (1.503-2.376) | <0.001 |

*^[7]^*The fully adjusted model included fever-burden group, age, sex, SOFA score, heart rate, respiratory rate, SpO2, white blood cell count, INR, coronary heart disease, atrial fibrillation, hypertension, mechanical ventilation, haemopurification, and temperature measurement density, with diabetes and race included as stratification variables. The no fever-burden group was used as the reference.

**Supplementary Material Table 7** Sensitivity analyses using 24-hour and 48-hour fever-burden windows

| **Window** | **Model** | **N** | **Events** | **Comparison** | **HR (95% CI)** | **P value** |
| --- | --- | --- | --- | --- | --- | --- |
| 24-hour fever-burden window | Crude model | 2,663 | 525 | Low vs none | 1.913 (1.468-2.492) | <0.001 |
| 24-hour fever-burden window | Crude model | 2,663 | 525 | High vs none | 2.119 (1.648-2.725) | <0.001 |
| 24-hour fever-burden window | Fully adjusted model | 2,663 | 525 | Low vs none | 1.418 (1.080-1.862) | 0.012 |
| 24-hour fever-burden window | Fully adjusted model | 2,663 | 525 | High vs none | 1.209 (0.918-1.594) | 0.177 |
| 48-hour fever-burden window | Crude model | 2,738 | 535 | Low vs none | 2.046 (1.621-2.583) | <0.001 |
| 48-hour fever-burden window | Crude model | 2,738 | 535 | High vs none | 2.608 (2.105-3.231) | <0.001 |
| 48-hour fever-burden window | Fully adjusted model | 2,738 | 535 | Low vs none | 1.504 (1.183-1.912) | <0.001 |
| 48-hour fever-burden window | Fully adjusted model | 2,738 | 535 | High vs none | 1.620 (1.276-2.055) | <0.001 |

^[8]^Hazard ratios were estimated using Cox proportional hazards models. The fully adjusted model included age, sex, SOFA score, heart rate, respiratory rate, SpO2, white blood cell count, INR, coronary heart disease, atrial fibrillation, hypertension, mechanical ventilation, and haemopurification or renal replacement therapy, with stratification by diabetes and race where appropriate. The reference group was no fever burden.

**Supplementary Material Table 8** Day-3 landmark analysis for 72-hour fever burden

| **Model** | **Group** | **HR (95% CI)** | **P value** |
| --- | --- | --- | --- |
| Crude model | Low fever burden | 2.224 (1.751-2.825) | <0.001 |
| Crude model | High fever burden | 2.615 (2.077-3.292) | <0.001 |
| Fully adjusted model | Low fever burden | 1.562 (1.219-2.002) | <0.001 |
| Fully adjusted model | High fever burden | 1.943 (1.502-2.513) | <0.001 |

^[9]^This day-3 landmark analysis excluded patients who died or were censored within the first 72 hours after ICU admission. Follow-up started at day 3 and ended at day 28. The fully adjusted model included age, sex, SOFA score, heart rate, respiratory rate, SpO2, white blood cell count, INR, coronary heart disease, atrial fibrillation, hypertension, mechanical ventilation, and haemopurification, with diabetes and race included as stratification variables.

**Supplementary Material Table 9** Sensitivity analyses using alternative temperature thresholds

| **Threshold** | **Model** | **Group** | **HR (95% CI)** | **P value** | **N** | **Events** | **Positive cutoff** |
| --- | --- | --- | --- | --- | --- | --- | --- |
| 37.5°C | Crude model | Low fever burden | 1.642 (1.320-2.044) | <0.001 | 2,781 | 544 | 1.004 |
| 37.5°C | Crude model | High fever burden | 2.759 (2.274-3.347) | <0.001 | 2,781 | 544 | 1.004 |
| 37.5°C | Fully adjusted model | Low fever burden | 1.285 (1.026-1.611) | 0.029 | 2,781 | 544 | 1.004 |
| 37.5°C | Fully adjusted model | High fever burden | 1.831 (1.475-2.273) | <0.001 | 2,781 | 544 | 1.004 |
| 37.8°C | Crude model | Low fever burden | 2.158 (1.742-2.672) | <0.001 | 2,781 | 544 | 0.732 |
| 37.8°C | Crude model | High fever burden | 2.776 (2.273-3.390) | <0.001 | 2,781 | 544 | 0.732 |
| 37.8°C | Fully adjusted model | Low fever burden | 1.569 (1.256-1.959) | <0.001 | 2,781 | 544 | 0.732 |
| 37.8°C | Fully adjusted model | High fever burden | 1.948 (1.557-2.436) | <0.001 | 2,781 | 544 | 0.732 |
| 38.0°C | Crude model | Low fever burden | 1.862 (1.474-2.352) | <0.001 | 2,781 | 544 | 0.566 |
| 38.0°C | Crude model | High fever burden | 2.644 (2.143-3.261) | <0.001 | 2,781 | 544 | 0.566 |
| 38.0°C | Fully adjusted model | Low fever burden | 1.352 (1.063-1.720) | 0.014 | 2,781 | 544 | 0.566 |
| 38.0°C | Fully adjusted model | High fever burden | 1.827 (1.446-2.310) | <0.001 | 2,781 | 544 | 0.566 |
| 38.3°C | Crude model | Low fever burden | 2.152 (1.680-2.757) | <0.001 | 2,781 | 544 | 0.404 |
| 38.3°C | Crude model | High fever burden | 2.681 (2.124-3.384) | <0.001 | 2,781 | 544 | 0.404 |
| 38.3°C | Fully adjusted model | Low fever burden | 1.548 (1.197-2.002) | <0.001 | 2,781 | 544 | 0.404 |
| 38.3°C | Fully adjusted model | High fever burden | 2.009 (1.556-2.595) | <0.001 | 2,781 | 544 | 0.404 |

^[10]^ Fever burden during the first 72 hours after ICU admission was recalculated using alternative temperature thresholds of 37.5°C, 37.8°C, 38.0°C and 38.3°C. For each threshold, patients were categorized into no, low and high fever-burden groups using the median value among patients with positive fever burden. The fully adjusted model included age, sex, SOFA score, heart rate, respiratory rate, SpO2, white blood cell count, INR, coronary heart disease, atrial fibrillation, hypertension, mechanical ventilation, and haemopurification, with diabetes and race included as stratification variables.

**Supplementary Material Table 10** Sensitivity analysis excluding patients with sepsis

| **Model** | **Group** | **HR (95% CI)** | **P value** |
| --- | --- | --- | --- |
| Crude model | Low fever burden | 3.160 (2.191-4.556) | <0.001 |
| Crude model | High fever burden | 6.371 (4.301-9.438) | <0.001 |
| Fully adjusted model | Low fever burden | 1.628 (1.103-2.402) | 0.014 |
| Fully adjusted model | High fever burden | 3.571 (2.292-5.565) | <0.001 |

^[11]^This sensitivity analysis excluded patients with sepsis. Fever burden was defined using the same 72-hour exposure window and the same fever-burden categories as in the primary analysis. The fully adjusted model included age, sex, SOFA score, heart rate, respiratory rate, SpO2, white blood cell count, INR, coronary heart disease, atrial fibrillation, hypertension, mechanical ventilation, and haemopurification, with diabetes and race included as stratification variables.

**Supplementary Material Table 11** Seven-day fever burden landmark analysis

| **Model** | **Group** | **HR (95% CI)** | **P value** |
| --- | --- | --- | --- |
| Crude model | Low fever burden | 2.619 (1.981-3.463) | <0.001 |
| Crude model | High fever burden | 3.323 (2.545-4.340) | <0.001 |
| Fully adjusted model | Low fever burden | 1.724 (1.283-2.317) | <0.001 |
| Fully adjusted model | High fever burden | 2.372 (1.744-3.227) | <0.001 |

^[12]^This supplementary analysis used fever burden during the first 7 ICU days and was performed as a day-7 landmark analysis. Patients who died or were censored within the first 7 days were excluded. Follow-up started at day 7 and ended at day 28. The fully adjusted model included age, sex, SOFA score, heart rate, respiratory rate, SpO2, white blood cell count, INR, coronary heart disease, atrial fibrillation, hypertension, mechanical ventilation, and haemopurification, with diabetes and race included as stratification variables.

***Abbreviations***: *CI, confiden*ce in*terval; HR, hazard ratio; ICU, intensive care unit; INR, international normalised ratio; SOFA, Sequential Organ Failure Assessment; SpO2, peripheral oxygen saturation; WBC, white blood cell count.*
